# On Vaccination

**Published:** 1804-06-01

**Authors:** G. C. Jenner


### To Dr. BRADLEY.

Sir,

EVERY effort to extend the practice of vaccination is certainly laudable. The obligation conferred on society by those who labour to promote it, is beyond the bounds of calculation. But, Sir, the old adage, *est modus in rebus,*

 should should be held in view, by all who address the public on this important subject. The pages of your excellent Journal are not confined to medical eyes only. As they have been the great vehicle of information, respecting the Jennerian discovery, they are to be found in the apartments of the mother, who wishes to screen her offspring from the horrors of the small-pox, as well as the surgeon or physician: Surely, then, she must turn with a sickened eye from the first paper inserted in your Journal, for the present month; besides, this paper contains no information which has not been given before. If you will turn to Dr. Jenner's first publication on the variolæ vaccinæ, you will there find (page 6) the following sentence: "During the progress of the disease, the lips, nostrils, eye-lids, and other parts of the body, are sometimes affected with sores; but these evidently arise from their being *rubbed or scratched with the patient's infected fingers.*" This part of the history of the cow-pox was undoubtedly mentioned, not only to point out a law in the agency of the vaccine matter on the animal œconomy, but to guard the inoculator against surprize at an occurrence similar to that which came under the observation of Mr. Coley; and I feel assured, both you and that gentleman will pardon the liberty I take in this suggestion*. I am, &c.

*May* 14, 1804. G. C. JENNER.

* The public have a reliance on receiving, through the medium of our Journal, every kind of information respecting the progress and effects of this important discovery; and Mr. Coley's case, we believe, will *co-operate* with Dr. Jenner's observations in *guarding* mothers from beholding " with a sickened eye," Mr. C's paper, because he has developed the true source of these unusual appearances. ED.

